# Microbiota composition and intestinal barrier function modulated by tamsulosin and *Lactococcus lactis* in a cirrhosis rat model

**DOI:** 10.3389/fcimb.2025.1624065

**Published:** 2025-09-02

**Authors:** Silvia Valeria Padilla-García, Abraham Loera-Muro, Martín Humberto Muñoz-Ortega, David Alejandro Hernández-Marín, Javier Ventura-Juárez, Sandra Luz Martínez-Hernández

**Affiliations:** ^1^ Department of Morphology, Center for Basic Sciences, Autonomous University of Aguascalientes, Aguascalientes, Ags, Mexico; ^2^ Secihti-Center for Biological Research of the Northwest, SC, La Paz, Mexico; ^3^ Department of Chemistry, Center for Basic Sciences, Autonomous University of Aguascalientes, Aguascalientes, Ags, Mexico; ^4^ Department of Microbiology, Center for Basic Sciences, Autonomous University of Aguascalientes, Aguascalientes, Ags, Mexico

**Keywords:** tamsulosin, *L. lactis*, microbiota, intestinal permeability, bacterial translocation, cirrhosis

## Abstract

**Introduction:**

The pathological progression of cirrhosis disrupts the gut-liver axis. *Lactococcus lactis* (*L. lactis*) exhibits immunomodulatory properties and an ability to enhance intestinal barrier function. It has been demonstrated that tamsulosin has antifibrotic and anti-inflammatory effects in hepatic injury models. This study evaluated the effect of a tamsulosin and *L. lactis* co-treatment on the recovery of microbiota and intestinal barrier integrity in a Wistar rat liver cirrhosis model.

**Material and methods:**

Male Wistar rats were administered CCl_4_ intraperitoneally for 4 weeks. Subsequently, rats received tamsulosin, *L. lactis*, or both, orally for 2 weeks. The intestinal microbiota was assessed using 16S rRNA gene sequencing. Intestinal barrier integrity was evaluated using qPCR and Western blot for proteins ZO-1, occludin, and claudin-2. Bacterial translocation was evaluated by endotoxin concentration, bacterial DNA, and microbial culture of extraintestinal tissues. Finally, hepatic, intestinal histology, and liver function markers were analyzed.

**Results:**

*L. lactis* and its combination with tamsulosin (T/*L. lactis*) increased microbial diversity and promoted a balanced gut microbiota characterized by a *Firmicutes* predominance followed by *Proteobacteria* and reduced *Clostridia* and *Gammaproteobacteria* levels. *L. lactis* group upregulated ZO-1 and occludin expression, while no significant changes were observed with tamsulosin or T/*L. actis* groups, nonetheless, intestinal morphology resembled that of healthy controls. Bacterial translocation analysis revealed no endotoxins, bacterial DNA, or bacteria in extraintestinal tissues. Both treatments also improved hepatic and intestinal histology, with partial liver function recovery.

**Conclusión:**

Findings such as reduced bacterial translocation, lower systemic endotoxin levels, improved intestinal morphology, and modulation of gut microbiota composition suggest that both agents (*L. lactis* and tamsulosin), particularly in combination, exert positive effects on the intestinal barrier in cirrhosis.

## 1 Introduction

Hepatic cirrhosis, is a chronic progressive disease with significant systemic consequences, arising from various hepatic insults that trigger inflammation and fibrosis. Its complications range from mild to severe, potentially leading to multi-organ failure and death (Roehlen et al., 2020). Cirrhosis develops after prolonged injury, which leads to the replacement of healthy liver parenchyma with fibrotic tissue and regenerative nodules, ultimately causing portal hypertension. The disease progresses from an asymptomatic stage (compensated cirrhosis) to a symptomatic one (decompensated cirrhosis), whose complications frequently require hospitalization (Ginès et al., 2021). The intestine is among the hepatic cirrhosis-impacted organs, where increased intestinal permeability leads to significant modifications in gut microbiota composition (Lee and Suk, 2020), as well as changes in the quantity and quality of mucus lining the intestinal epithelium, along with dysmotility and damage to the epithelial barrier. Cirrhotic patients exhibit an overgrowth of potentially pathogenic bacteria accompanied by a reduction in autochthonous bacterial populations (Fukui, 2021). Pathological bacterial translocation plays a critical role in the onset of various infections. In cirrhotic patients, it contributes to the development of spontaneous bacterial peritonitis and is associated with multi-organ failure in critically ill individuals, as well as progressive hepatic hemodynamic deterioration (Garcı、a-Tsao, 2001; Wiest et al., 2014). Gut alterations associated with liver cirrhosis arise from the bidirectional interaction and complex crosstalk between the gut and the liver (Albillos et al., 2020). Recent studies have explored the use of various probiotic supplements as potential therapeutic interventions for cirrhosis, with some reporting beneficial effects in affected patients. *L. lactis* is a Gram-positive, spherical, homolactic, non-sporulant, and facultative anaerobic bacterium (Parapouli et al., 2013). It plays a relevant role in the intestine as an immunomodulator by promoting the production of anti-inflammatory cytokines, strengthening the intestinal barrier, and competing against pathogens (Laguna et al., 2024). Previous studies from our group have reported that oral administration of *L. lactis* may serve as a potential strategy to prevent and protect against liver injury. *L. lactis* attenuated hepatic cirrhosis by preventing steatosis and fibrosis, decreasing serum aspartate aminotransferase (AST) and alanine aminotransferase (ALT) levels, downregulating hepatic IL-1β, and enhancing anti-inflammatory responses through increased expression of Foxp3 and IL-10 (Delgado-Venegas et al., 2021).

On the other hand, tamsulosin is a selective α1-adrenergic antagonist with a prolonged therapeutic effect, commonly prescribed for the management of genitourinary conditions. It primarily acts on the smooth muscle of the urethra, bladder neck, and prostate, inducing muscle relaxation and thereby improving urinary flow and reducing symptoms associated with lower urinary tract obstruction (Dunn et al., 2002; Song et al., 2020). Pharmacological inhibition using nonselective α- and β-adrenergic receptor antagonists has been shown to suppress hepatic stellate cell (HSC) proliferation and attenuate fibrogenesis (Liu et al., 2009). Studies have shown that tamsulosin facilitates improved recovery of hepatic architecture, accompanied by a reduction in fibrosis and NF-κB activation (Soleil et al., 2022) in cirrhotic rats. Primary HSCs were found to express adrenoreceptors α-1B, α-1D, β1, and β2 by RT-PCR analysis. Neurotransmitters (noradrenaline and adrenaline) stimulate and activate hepatic stellate cells by binding to cells through α1-adrenergic receptors, triggering a cellular response that leads to the release of inflammatory cytokines (Oben and Diehl, 2004; Sigala et al., 2013). Previous studies have shown that the α1-adrenoceptor blocker doxazosin decreases the fibrogenic activity of activated HSCs, which is associated with the induction of cellular senescence via α1-AR antagonism, suggesting that α1-AR is a potential treatment for liver fibrosis. The adrenoblocker downregulated collagen I and ACTA2 and increased PPAR-γ expression both in the presence and absence of TGF-β, confirming that doxazosin delays stellate cell activation (Serna-Salas et al., 2022). Tamsulosin also directly modulates the HSC, reducing quiescent and activated forms (Buendía-Delgado et al., 2022) and promoting anti-inflammatory processes (Alabdali and Ibrahim, 2023; Dunn et al., 2002; Song et al., 2020).

To date, no studies have investigated the effects of tamsulosin on intestinal barrier permeability or gut microbiota composition in experimental cirrhosis models. Furthermore, it remains unknown whether co-administration of this drug with *L. lactis* may enhance intestinal integrity, influence bacterial translocation, and thereby mitigate the effects of cirrhosis.

## 2 Materials and methods

### 2.1 Animal conditions

Wistar rats were obtained from the Central Bioterium of the Autonomous University of Aguascalientes and kept in the local Animal Core Facility at 25°C with 12-h light/dark cycles. The rats were fed Laboratory Chow (Purina, Mexico) and provided tap water ad libitum. All animals were treated with fenbendazole (55 mg), toltrazuril (20 mg), and praziquantel (10 mg) (1 ml/kg) for 3 days. Finally, animals were kept under standard and hygienic conditions to acclimate for 7 days before starting the cirrhosis induction.

### 2.2 *Lactococcus lactis* strain and culture conditions

*L. lactis* strain NZ9000 was grown in 10% glucose-M17 broth (BD Difco, Sparks, MD, USA) at 30°C. Cells were harvested at 8,000 *xg* for 5 min. Finally, cells suspensions were adjusted to 1×10^9^ CFU/ml in sterile water based on an optical density (OD) at 600 nm of 1.0. Doses of 1×10^9^ CFU in 250 µl per animal were prepared from fresh cultures.

### 2.3 Experimental design

**Figure 1:**
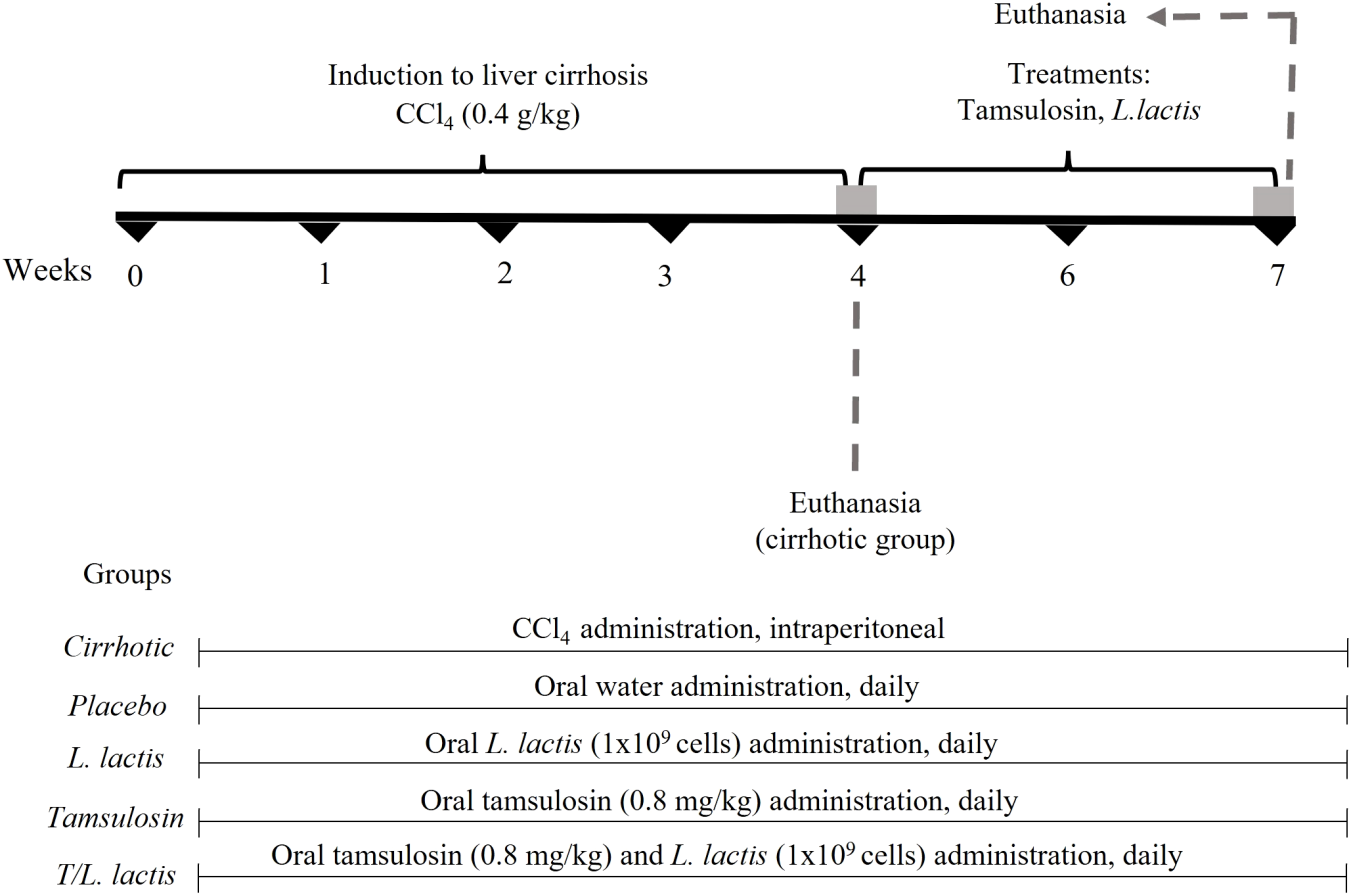
Treatment protocol with tamsulosin and *L. lactis*. CCl_4_ (carbon tetrachloride) was administered intraperitoneally at a dose of 0.4 g/kg, three times per week for 4 weeks. Tamsulosin hydrochloride (tamsulosin) (0.8 mg/kg/day) and *L. lactis* (NZ9000) (1×10^9^ cells/ml/day) were administered orally for 2 weeks. At the end of the treatment period, all animals were euthanized, and tissue samples were collected.

A total of 30 male Wistar rats (150–200 g) were randomly divided into 6 groups (n=5): (*i*) control (healthy), (*ii*) cirrhotic (diseased), (*iii*) placebo (endogenous recovery), (*iv*) *L. lactis* (treated with *L. lactis*), (*v*) tamsulosin (treated with tamsulosin), (vi) T/*L. lactis* (treated with tamsulosin and *L. lactis*). To induce liver cirrhosis, carbon tetrachloride (CCl_4_, Sigma-Aldrich, Darmstadt, Germany) was administered intraperitoneally at a 0.4 g/kg dose, three times per week for four weeks (Macías-Pérez et al., 2019). Once the induction was completed, treatments were started. Orally, for 2 weeks, 0.8 mg/kg/day of tamsulosin hydrochloride (Pharmalife LTC) and *L. lactis* (1×10^9^ CFU in 250 µl) were suspended in sterile purified water and administered separately once daily, using a curved stainless steel esophageal cannula (18x3 mm, Cadence Science). At the end of the treatments, all rats were euthanized with sodium pentobarbital overdose (≥100 mg/kg) applied intraperitoneally, until rapid loss of consciousness, thus minimizing the stress and anxiety experienced by the animal, while monitoring respiratory and cardiac signs until their absence. All animal experiments were approved by the Ethics Committee for the use of animals in teaching and research at UAA (CEADI−UAA, UAA: Autonomous University of Aguascalientes, AUT−B−C−1121−077), following the Mexican Official Standard NOM−062−ZOO−1999 (Muñoz-LIO, 2001) and the guidelines of the National Institutes of Health for the care and use of Laboratory animals (National Research Council, 2011) samples were taken from the portal vein and by cardiac puncture, and under sterile conditions, samples of intestinal tissue, liver, mesenteric lymph node (MLN), and spleen were collected for further analysis. Additionally, fecal samples were directly collected from the intestine and stored at -80°C until processing (**Figure 1**).

### 2.4 Analysis of intestinal bacterial community

Fecal samples were collected at the end of the study and placed in sterile containers. Bacterial DNA was isolated using the Quick-DNA™ Fecal/Soil Microbe Miniprep Kit (Zymo Research, OC, California) according to the manufacturer’s specifications. The DNA pellet was resuspended in 50 µl of ultra-pure distilled water. The integrity of total DNA (100–200 ng) for each sample was verified by 1.0% agarose gel electrophoresis, and its quality was assessed using a Bio-Drop-LITE spectrophotometer (Isogen Lifescience, UT, Netherlands). The extracted DNA was stored at -80°C until sequencing. From the total extracted DNA, the V3 region of the 16S rDNA gene in prokaryotes (bacteria and archaea) was amplified using the V3-338F (5′-ACTCCTACGGGAGGCAGC-3′) and V4-806R (5′ GGACTACHVHHHTWTCTAAT-3′) primers. The amplified regions were sequenced on the Illumina MiniSeq platform 2×150 (300 cycles) (Illumina) at the Centro de Investigación en Alimentos y Desarrollo (CIAD, Mazatlan, Sinaloa, Mexico). Raw reads were filtered, trimmed, and dereplicated; paired-end reads were merged, denoised, and chimeras were removed using the DADA2 (version 1.8) pipeline within QIIME2 (version 2020.2). Taxonomic assignment of amplicon sequence variants (ASVs) was performed using the SILVA-132 database. Sequences assigned to eukaryotes and archaea were discarded. Alpha diversity metrics were calculated, including relative taxon abundance, Shannon and Simpson diversity indices, and Pielou’s evenness index. Additionally, the UniFrac distance matrix was computed (QIIME2, version 2020.2).

### 2.5 Intestinal permeability: ZO-1, occludin and claudin-2 determination

The effects of treatments with tamsulosin and *L. lactis* on the mRNA and protein levels of ZO-1, occludin, and claudin-2 were evaluated using quantitative PCR (qPCR) and Western Blot analysis, respectively. For qPCR analysis, total RNA was extracted using the Direct-zol™ RNA MiniPrep Kit (Zymo Research), following the manufacturer’s instructions. The RNA was quantified using the BioDrop (Isogen Life Science B.V.). For cDNA synthesis, reverse transcription was performed with 1 µg of RNA using the GoScript™ Reverse Transcription System (Promega). Subsequently, qPCR was performed using the Maxima SYBR Green/ROX qPCR Master Mix (2X) (Thermo Fisher Scientific) with the StepOne™ Real-Time PCR System (Applied Biosystems). The primers for the ZO-1, occludin, claudin-2, and *β*-actin genes are shown in **Table 1**. Thermocycling conditions were as follows: 50°C for 2 min, 95°C for 3 min, followed by 40 cycles of 95°C for 40 s and 60°C for 45 s. The relative expression levels were normalized to β-actin, and differences were determined using the 2^−ΔΔCq^ method (Livak and Schmittgen, 2001). The specificity of the PCR product was confirmed by gel electrophoresis (1.0%). For Western blot analysis, intestinal samples were lysed on ice in RIPA buffer (Pierce RIPA buffer, Thermo Scientific). After homogenization and centrifugation at 14,000 rpm for 15 min at 4°C, protein concentration was measured using a standard Bradford assay (Bio-Rad). 50 μg of total protein was separated on polyacrylamide gels (30% acrylamide/bis-acrylamide) at 4% and 12% (Bio-Rad) and transferred to a PVDF membrane (Bio-Rad). The membranes were incubated with anti-rat ZO-1 (1:1000, Santa Cruz Biotechnology), anti-mouse occludin (1:300, Santa Cruz Biotechnology), anti-mouse claudin-2 (1:400, Santa Cruz Biotechnology), and anti-rabbit *β*-actin (1:10 000, Abcam) antibodies overnight at 4°C. Afterwards, the primary antibody was removed, and the membranes were re-incubated with secondary antibodies: goat anti-mouse IgG (HRP, 1:5000, Thermo Fisher Scientific) and anti-rat IgG (HRP, 1:10,000, Abcam). The blots were developed with the Clarity Western ECL substrate (Bio-Rad) to obtain chemiluminescence images.

**Table 1:** Oligonucleotides used in this study.

Oligonucleotide	Sequence	Tm
ZO-1	FW: ATTCAGTTCGCTCCCATGAC RW: GCTGTGGAGACTGTGTGGAA	58°C
Ocludina	FW: AGGACAGACCCAGACCACTA RW: ACTCTTCGCTCTCCTCTCTG	58°C
Claudina-2	FW: AAGGTGCTGCTGAGGGTAGA RW: CATAGCAAAAAGTGGCAGCA	57°C
16s	FW: TCCTACGGGAGGCAGCAGT RW: GGACTACCAGGGTATCTAATCCTGTT	58°C
β-Actina	FW: GTCGTACCACTGGCATTGTG RW: GCTGTGGTGGTGAAGCTGTA	62°C

### 2.6 Bacterial translocation assay

Tissues from MLN, spleen, and liver, previously obtained, were homogenized under sterile conditions. 100 µl of the homogenate from each tissue was collected and inoculated into different tubes containing M17 broth (BD Difco, Sparks, MD, USA), brain-heart broth (BD Bioxon, Mexico), thioglycolate (BD Bioxon, Mexico), and standard count agar (BD Bioxon, Mexico). Subsequently, they were plated onto selective media such as MacConkey, mannitol salt, and blood agar. After 48 hours of incubation at 37°C, the number of viable colonies on each plate was counted, and specific microorganisms were identified through biochemical tests. The positivity observed in these organs was considered indicative of the passage of bacteria to the portal or systemic circulation. On the other hand, bacterial DNA was determined using 1 ml of blood extracted from the portal vein. Blood was processed using the Quick-DNA™ Miniprep Plus kit (Zymo Research, OC, California), following the manufacturer’s instructions. DNA concentration was determined using the Bio-Drop-LITE spectrophotometer (Isogen Lifescience, UT, Netherlands). The 16S rDNA region was amplified by PCR in a thermal cycler (Swift MiniPro, Singapore). The PCR thermal cycles were as follows: 95°C for 3 min, 95°C for 45 s, 58°C for 35 s, 72°C for 12 s, and 72°C for 3 min. The primers set are shown in **Table 1**. Furthermore, endotoxin levels were measured through the LAL assay (Pierce Chromogenic Endotoxin Quantification Kit, Thermo Scientific, Waltham, MA, USA) as per the manufacturer’s instructions. The concentration of endotoxin (EU/ml) was calculated using a standard curve, and the absorbance of each well was measured at 450 nm using a microplate reader.

### 2.7 Histopathological analysis

Intestinal and liver samples were evaluated using hematoxylin-eosin (H&E) staining to assess architectural and morphological tissue changes. Sirius Red staining, observed under polarized light microscopy, was used to identify collagen fiber deposits (type I = red, type III = green). Tissue samples were fixed in 4% paraformaldehyde (PFA) for 24–48 hours before paraffin embedding. Serial sections (5 µm thick) of paraffin-embedded tissue were cut using a microtome (Leica RM 2125RT). Paraffin sections were then deparaffinized by heating at 60°C, washed with xylene as the deparaffinizing agent, and rehydrated through a graded ethanol series before staining. Histological preparations were visualized using a Zeiss Axioscope 40/40 FL microscope and analyzed with Image Pro Plus Software 4.5.1 (Media Cybernetics, Bethesda, MD, USA). For intestinal tissue, histomorphometric parameters were assessed in H&E-stained sections by measuring villus height (5 villi per slice and 3 slices per sample) and crypt depth (5 crypts per slice and 3 slices per sample).

### 2.8 Markers of liver damage

Serum samples were analyzed to determine liver damage, with quantification of levels of ALT, AST, and alkaline phosphatase (AP) using a spectrophotometric semiautomatic BTS-350 analyzer (Biosystems, Quezon City, Philippines).

### 2.9 Statistical analysis

Statistical analysis was performed using GraphPad Prism 8.0.2 software (GraphPad Software, San Diego, CA, USA). Data are presented as mean ± standard error of the mean (SEM). Differences between groups were assessed using two-way analysis of variance (ANOVA), followed by Tukey’s *post hoc* test. For non-parametric data, a Kruskal–Wallis test was used for multiple group comparisons, followed by Dunn’s *post hoc* test, or a Mann–Whitney U test for comparisons between two groups. A *p*-value < 0.05 was considered statistically significant. PERMANOVA analysis was performed using the ‘adonis’ function from the ‘vegan’ package.

## 3 Results

### 3.1 Effect of tamsulosin and *L. lactis* on intestinal barrier integrity

#### 3.1.1 Intestine histological changes

**Figure 2:**
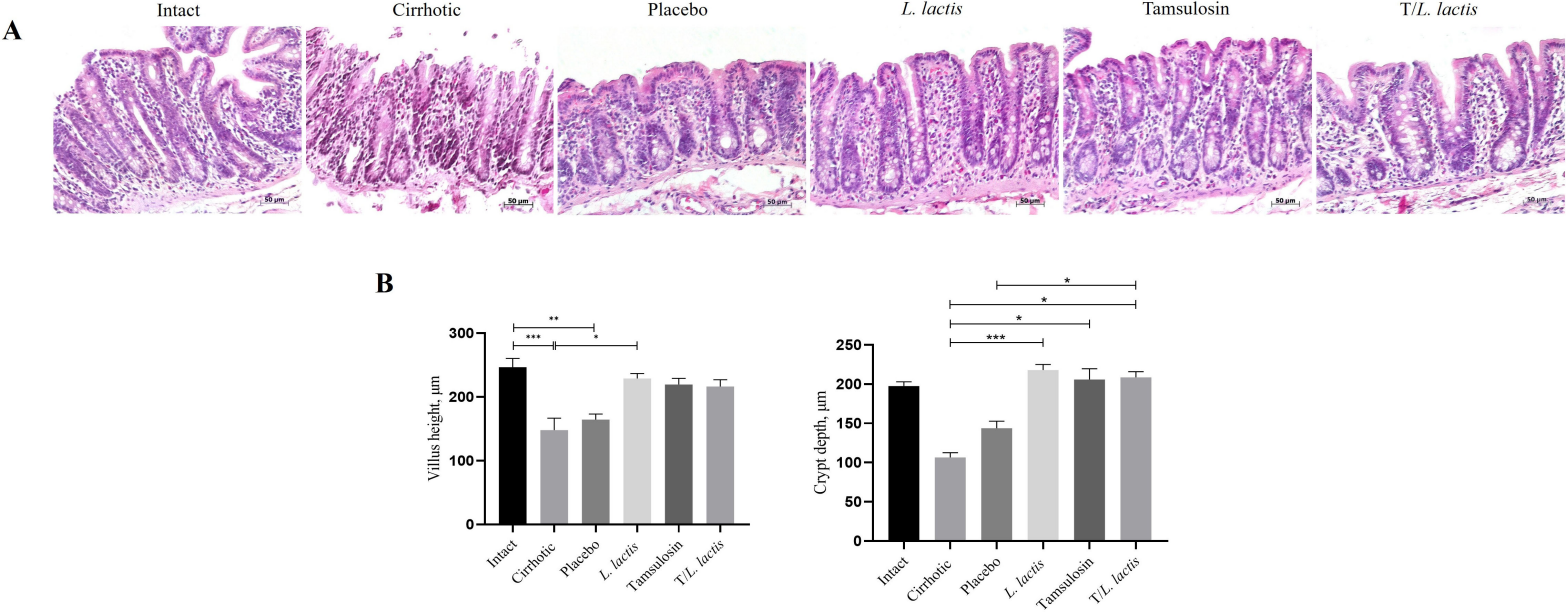
Analysis of intestinal morphology. **(A)** Representative histological images were obtained at a magnification of 20x. **(B)** Analysis of intestinal villus height and crypt depth. The results are presented as mean ± standard error of the mean. Samples from each rat were analyzed in triplicate. The statistical analysis was performed with the Kruskal-Wallis and Tukey post-test methods, where the values of *p < 0.05, **p < 0.01, and ***p < 0.001 were considered significant.

In the histopathological analysis performed on intestinal samples, the intact group displayed a mucosa with a surface composed of normal goblet cells, abundant crypts, mucous cells, and microvilli. Additionally, a characteristic lamina propria was observed, rich in lymphoid cells, bordered by the muscularis mucosae. In the cirrhotic group, a thinning of both the epithelium and lamina propria was noted, along with a loss of the brush border structure and a reduction in goblet cells (**Figure 2A**). Villus height and crypt depth were reduced (147.95 µm and 106.50 µm, respectively) compared to the intact group (246.58 µm, *p* < 0.001 and 197.6 µm, respectively). The placebo group exhibited a more uniform structure; however, villus height (164.2 µm) and crypt depth (143.8 µm) were also reduced. Intestinal morphology of the *L. lactis*, tamsulosin, and T/*L. lactis* groups closely resembled that of the intact group, with villus height (x̅, 221.49 µm) and crypt depth (x̅, 210.88 µm) remaining unchanged across all three groups (**Figure 2B**).

#### 3.1.2 mRNA and protein expression of ZO-1, occludin and claudin-2 in intestinal tissue

**Figure 3:**
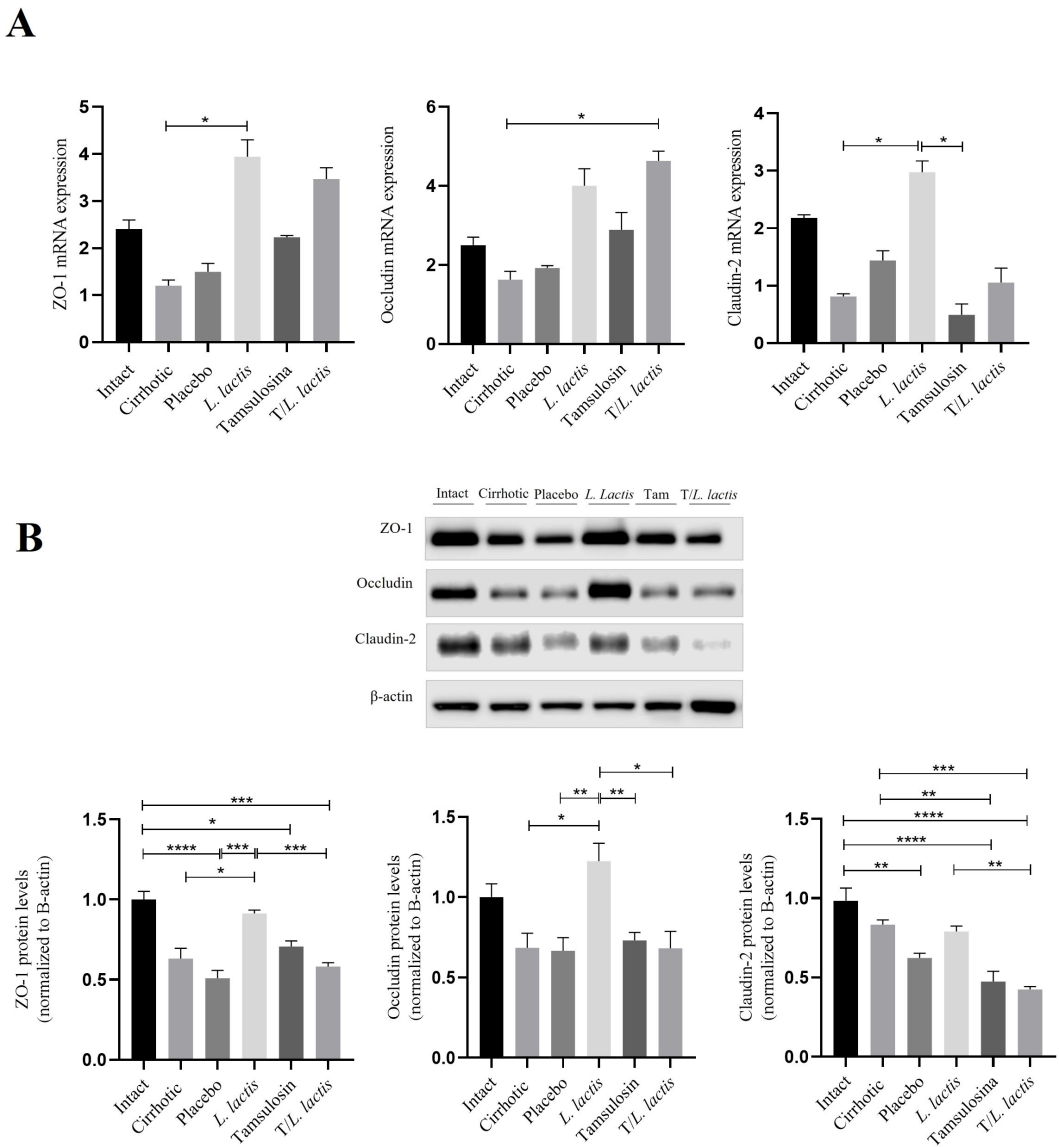
Intestinal Barrier: mRNA and Protein Expression Analysis. **(A)** mRNA of ZO-1, occludin, and claudin-2. **(B)** Densitometric analysis of ZO-1, occludin, and claudin-2 in intestinal tissue. Data corresponds to the mean ± SEM of three independent experiments. The statistical analysis was performed with the Kruskal-Wallis and Tukey post-test methods, where the values of *p < 0.05, **p < 0.01, ***p < 0.001, and ****p < 0.0001 were considered significant.

RT-qPCR and Western blot analyses were performed to evaluate the effect of tamsulosin and *L. lactis* on the ZO-1, occludin, and claudin-2 expression. The mRNA levels of ZO-1, occluding, and claudin-2 were reduced in the cirrhotic (1.02-fold, 1.62-fold, 0.81) and placebo (1.49-fold, 1.92-fold, 1.43-fold) groups, and their expression was lower than in control animals (2.40-fold, 2.50-fold, 2.17-fold). Regarding the groups that received the different treatments, we observed that *L. lactis* enhanced the mRNA expression of ZO-1 (3.94-fold; *p* < 0.05: *L. lactis vs* cirrhotic) and claudin-2 (2.97-fold; *p* < 0.05: *L. lactis vs* cirrhotic). *T/L*. *lactis* showed significant differences in occludin expression (4.63-fold; *p* < 0.05: T/*L. lactis vs* cirrhotic). The tamsulosin group did not show changes in the expression of ZO-1, occludin, and claudin-2 (**Figure 3A**). Finally, densitometric analysis showed that ZO-1 protein expression exhibited significant differences only in the *L. lactis* group (0.9-fold; *p* < 0.05: *L. lactis vs* cirrhotic, *p* < 0.001: *L. lactis vs* placebo, *p* < 0.001: *L. lactis vs* T/*L. lactis*), although not reaching the levels seen in the intact (1.0-fold) group. Similarly, a significant increase in occludin expression was observed in the *L. lactis* group (1.22-fold; *p* < 0.05, *p* < 0.01) compared to the cirrhotic, placebo, tamsulosin, and T/*L. lactis* groups. As for claudin-2, no significant differences were observed among the treatment groups (**Figure 3B**).

#### 3.1.3 Bacterial translocation

**Figure 4:**
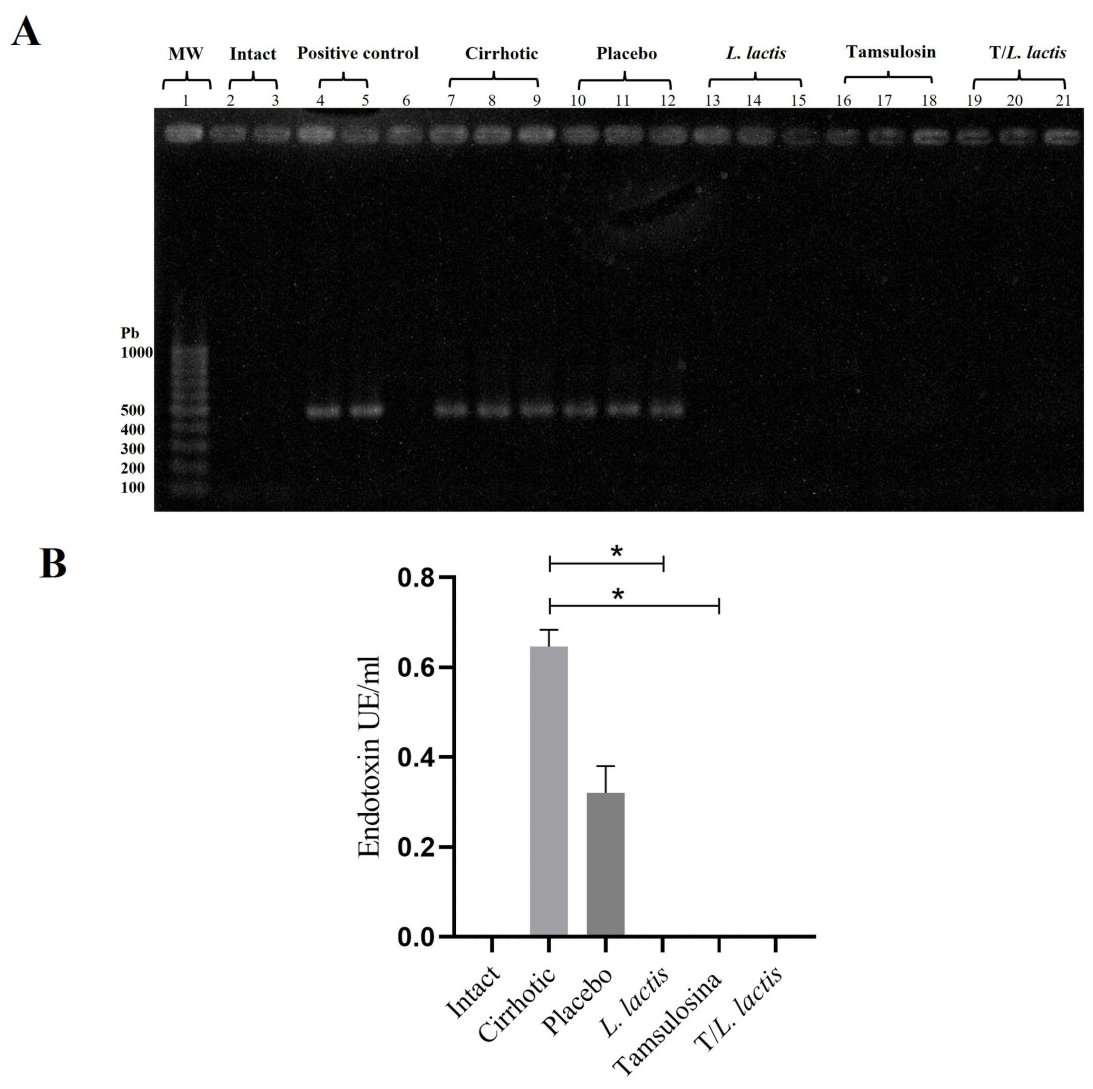
Effects on bacterial translocation. **(A)** PCR amplification of the 16S rRNA gene from bacterial DNA. **(B)** Serum endotoxin levels measured using a Kinetic Turbidimetric LAL assay. No amplification of the 16S rRNA gene and no detectable endotoxin levels were observed in the groups treated with tamsulosin, *L. lactis*, or their combination. The statistical analysis was performed using the Kruskal–Wallis and Tukey post-test methods, where the value of *p < 0.05 were considered significant.

The evaluation of bacterial translocation demonstrated the absence of bacterial growth in the intact group. In the cirrhotic and placebo groups, microorganisms were found in MLN, spleen, and liver. The isolated microorganisms were of enteric origin, with *Escherichia coli* being the most common (**Table 2**). In the *L. lactis*, tamsulosin, and T/*L. lactis* groups, no bacteria were detected in the MLN, spleen, or liver. On the other hand, blood samples processed for bacterial DNA identification showed the presence of the 16S gene in the cirrhotic and placebo groups. In the treatment groups, the 16S gene was not identified (**Figure 4A**). Finally, the results of plasma endotoxin measurement showed only concentrations of 0.65 EU/ml and 0.32 EU/ml in the cirrhotic and placebo groups, respectively. Animals treated with *L. lactis*, tamsulosin, and T/*L. lactis* did not show endotoxin concentrations (**Figure 4B**).

**Table 2:** Bacteria species isolated from mesenteric lymph nodes, spleen, and liver.

Group	MLN	Spleen	Liver
Intact	**_**	**_**	**_**
Cirrhotic	*E. coli* *E. agglomerans* *P. vulgaris*	*E. coli*	*E. coli*
Placebo	*E. coli*	*E. coli*	*E. coli*
*L. lactis*	**_**	**_**	**_**
Tamsulosin	**_**	**_**	**_**
T/*L. lactis*	**_**	**_**	**_**

*E. coli*, *Escherichia coli*; *P. mirabilis*, *Proteus mirabilis*; *P. Vulgaris*; *Proteus vulgaris*.

MNL, Mesenteric Lymph Node.

### 3.2 Effect of tamsulosin and *L. lactis* on gut microbiota composition

#### 3.2.1 Changes in gut bacterial diversity

**Figure 5:**
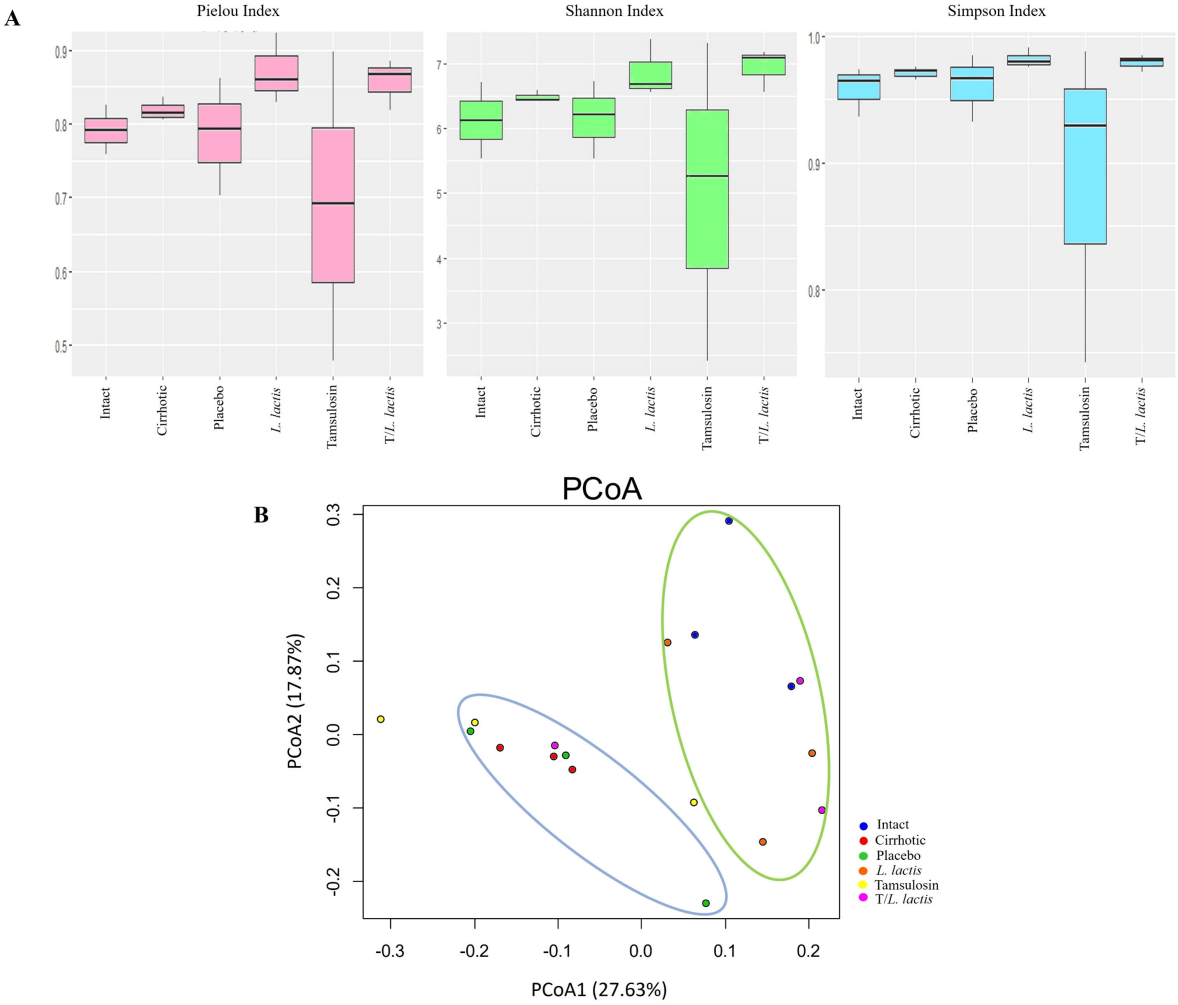
α and β diversity of gut microbiota between intact, cirrhotic, placebo*, L. lactis*, tamsulosin, and T/*L. lactis* groups presented by box plot. **(A)** Alpha diversity analysis showed that the *L. lactis* and T/*L. lactis* groups exhibited higher microbial evenness and diversity (Pielou, Shannon, and Simpson indices), while the tamsulosin group showed reduced values compared to the cirrhotic and placebo groups. **(B)** Beta diversity analysis, based on UniFrac distances and visualized by PCoA, revealed two distinct clusters: one (blue ellipse) comprising the cirrhotic, placebo, and tamsulosin groups, and a second cluster (green ellipse) grouping the intact, *L. lactis*, and T/*L. lactis* subjects. Similarities in microbial community composition were observed within each cluster, with clear differentiation between them.

Pielou’s evenness index revealed distinct patterns in microbial community structure among the analyzed groups. The *L. lactis* group exhibited higher microbial community evenness. T/*L. lactis* group showed slightly lower evenness, compared to the *L. lactis* group. The tamsulosin group presented a reduction in microbial community evenness compared with untreated groups (cirrhotic and placebo). According to the Shannon and Simpson indices, the *L. lactis* and T/*L. lactis* groups exhibited higher diversity levels. The tamsulosin group showed reduced microbial diversity compared with the cirrhotic and placebo groups (**Figure 5A**). Beta diversity was assessed using a UniFrac distance matrix and visualized via principal coordinate analysis (PCoA) to explore differences in microbial community composition. The PCoA revealed two distinct clusters: one cluster (blue ellipse) included the cirrhotic, placebo, and tamsulosin groups, indicating similar microbial community structures among them; the second cluster (green ellipse) comprised samples from the intact, *L. lactis*, and T/L. *lactis* groups, which exhibited greater similarity in microbial composition among themselves and clear differentiation from the cirrhotic group (**Figure 5B**).

#### 3.2.2 Changes in gut microbial community composition

**Figure 6:**
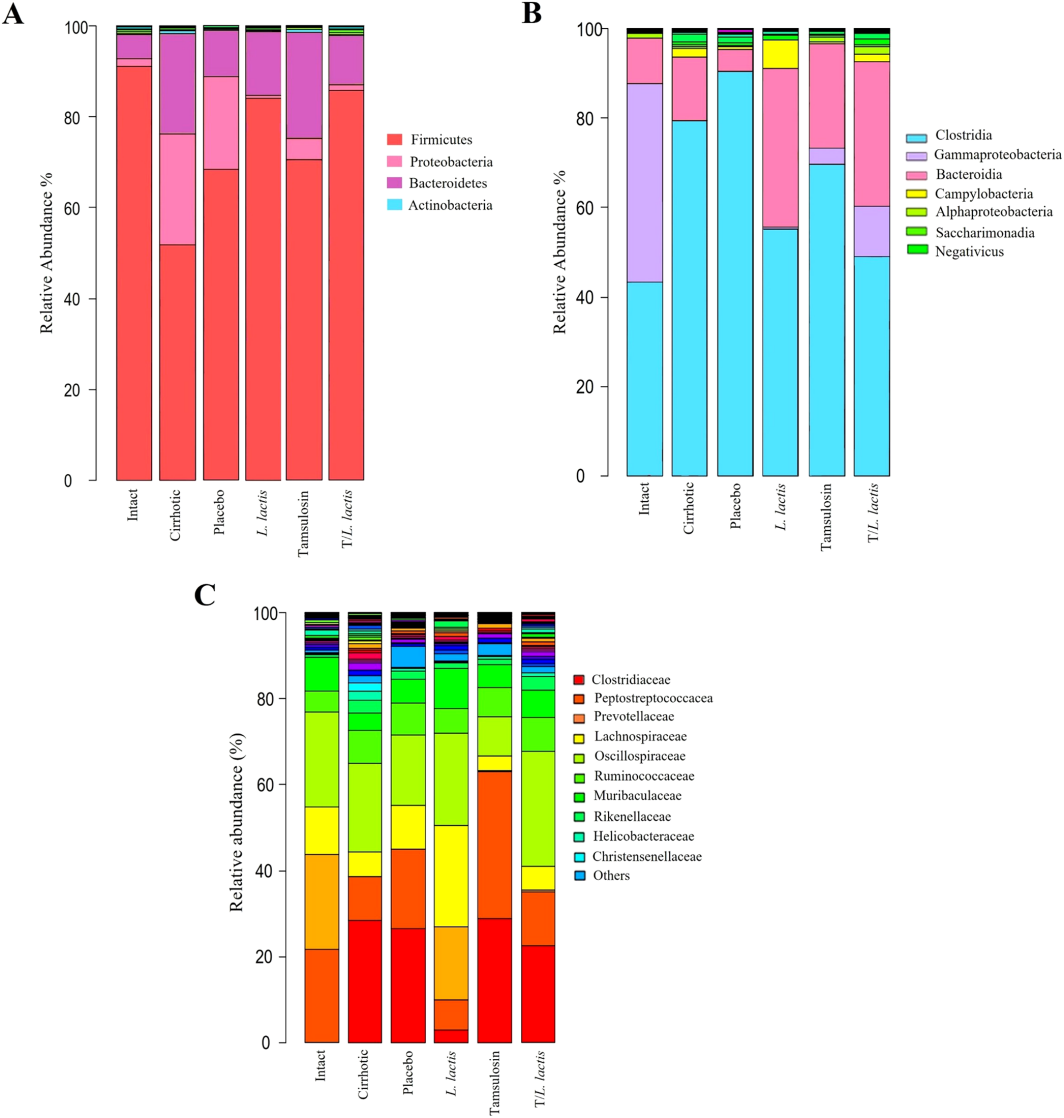
Change in intestinal microbial composition. Relative abundance bar plots of intestinal microbiota at the phylum, class, and family levels **(A)** *L. lactis* and T/*L. lactis* showed a dominance of *Firmicutes*, followed by *Proteobacteria*, and **(B)** reduced levels of *Clostridia* and Gammaproteobacteria were observed. **(C)** At the family level, *L. lactis* and T/*L*. *lactis* showed reduced abundances of *Clostridiaceae* and *Peptostreptococcaceae*, an increase of *Prevotellaceae* and *Lachnospiraceae*. Tamsulosin reduced *Firmicutes* and increased *Clostridia* and *Clostridiaceae* abundances.

The analysis of the relative abundance of bacterial populations in the different rat groups revealed phylum-level alterations in the cirrhotic group compared to the control group: *Firmicutes* (52.96% *vs*. 90.76%), *Proteobacteria* (24.47% *vs.* 2.12%), and *Bacteroidetes* (21.72% *vs.* 6.23%). The placebo group exhibited results similar to those of the cirrhotic group: *Firmicutes* (68.96%), *Proteobacteria* (21.47%), and *Bacteroidetes* (8.72%). Regarding the *L. lactis* group *vs*. the cirrhotic group: *Firmicutes* (84.32% *vs.* 52.96%), *Proteobacteria* (0.6% *vs.* 24.47%), and *Bacteroidetes* (14.28% *vs*. 21.72%). The tamsulosin group *vs*. the cirrhotic group showed the following relative abundances: *Firmicutes* (71.32% *vs*. 52.96%), *Proteobacteria* (4.65% *vs*. 24.47%), and *Bacteroidetes* (23.41% *vs*. 21.72%). Finally, for the T/*L. lactis* group *vs*. the cirrhotic group, the proportions were as follows: *Firmicutes* (87.68% vs. 52.96%), *Proteobacteria* (1.73% *vs*. 24.47%), and *Bacteroidetes* (10.12% *vs*. 21.72%). The proportion of *Firmicutes* in the cirrhotic group was lower compared to all other groups, while *Proteobacteria* and *Bacteroidetes* were found to be increased (**Figure 6A**). At the class level, *Clostridia* were predominant in all groups. In the control group, *Clostridia* showed a proportion of 43.12%, followed by *Gammaproteobacteria* (47.09%), *Bacteroidia* (8.31%), and other classes (1.48%). In the cirrhotic and placebo groups, there was a predominant increase in *Clostridia* (77.13% and 87.94%, respectively) and *Bacteroidia* (14.64% and 4.87%, respectively). In the group treated with *L. lactis*, *Clostridia* were reduced (56.21%) compared to the other groups, except for the T/*L. lactis* group (48.76%). *Gammaproteobacteria* were found at 0.3%, *Bacteroidia* at 32.60%, and *Campylobacteria* at 8.15%. In the tamsulosin and the T/*L. lactis*-treated groups, the proportion of *Clostridia* were 74.45% and 48.76%, respectively; *Gammaproteobacteria* were 2.83% and 14.23%, and *Bacteroidia* were 16.87% and 28.98%, respectively (**Figure 6B**). Finally, family-level analysis revealed the absence of *Clostridiaceae* but the presence of *Peptostreptococcaceae* (21.12%) *Prevotellaceae* (22.09%), *Lachnospiraceae* (9.03%) *Oscillospiraceae* (10.14%), and *Ruminococcaceae* (3.33%) in the control group. In the cirrhotic and placebo groups, an increase in *Clostridiaceae* (27.45% and 25.61%, respectively) was observed, along with a decrease in *Peptostreptococcaceae* (9.02%,19.56%) and *Lachnospiraceae* (6.31% and 10.04%). In the *L. lactis* treated group, *Clostridiaceae* (3.17%) and *Peptostreptococcaceae* (7.45%) decreased, while *Prevotellaceae* (28,32%)*, Lachnospiraceae* (22.78%) and *Oscillospiraceae* (20.01%) increased. The tamsulosin group showed an increase in *Clostridiaceae* (27.27%), and *Peptostreptococcaceae* (37.11%) was found in higher proportions compared to the other groups, whereas *Lachnospiraceae* (2.50%) and *Oscillospiraceae* (9.98%) exhibited reduced relative abundances. The T/*L. lactis* group exhibited a decrease in *Clostridiaceae* (0.23%) and *Peptostreptococcaceae* (3.42%). *Prevotellaceae* (17.11%)*, Lachnospiraceae* (10.15%)*, and Oscillospiraceae* (13.61%) exhibited higher abundance (**Figures 6C**, **Table 3**).

**Table 3:** Relative abundance (%) of major microbial taxa identified by 16S rRNA sequencing.

Taxonomic Group	Intact	Cirrhotic	Placebo	*L. lactis*	Tamsulosin	T/*L. lactis*
Phylum						
*Firmicutes*	90.76%	52.96%	68.96%	84.32%	71.32%	87.68%
*Proteobacteria*	2.12%	24.47%	2147%	0.6%	4.65%	1.73%
*Bacteroidetes*	6.23%	21.72%	8.72%	14.28%	23.41	10.12%
Class						
*Clostridia*	43.12%	77.13%	87.94%	56.21%	74.45%	48.76%
*Gammaproteobacteria*	47.09%	–	–	0.3%,	2.83%	14.23%
*Bacteroidia*	8.31%	14.64%	4.87%	32.60%	16.87%	28.98%
Family						
*Clostridiaceae*	–	27.45%	25.61%	3.17%	27.27%	0.23%
*Peptostreptococcaceae*	21.12%	9.02%,	19.56%	7.45%	37.11%	3.42%
*Prevotellaceae*	22.09%	–	–	28.32%	–	17.11%
*Lachnospiraceae*	9.03%	6.31%	10.04%	22.78%	2.50%	10.15%
*Oscillospiraceae*	10.14%	20.66%	17.81%	20.01%	9.98%	13.61%

**Figure 7:**
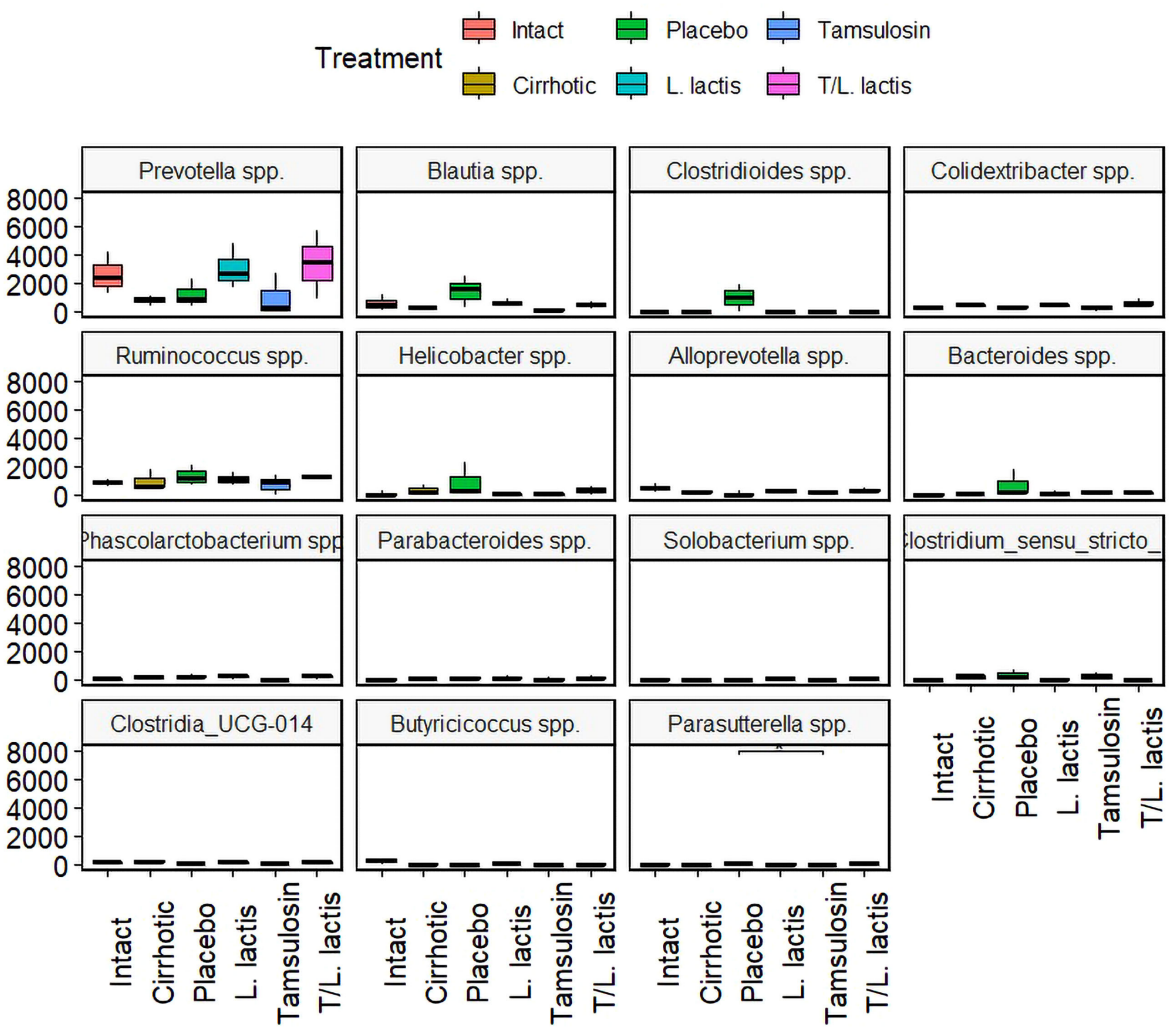
Change in intestinal microbial composition. Relative abundance box plot of intestinal microbiota at the genus level. *Prevotella* spp., showed higher abundance in the *L. lactis* and T/*L. lactis* groups. In contrast, the placebo group exhibited elevated levels of *Blautia* spp., *Clostridioides* spp., *Helicobacter* spp., *Bacteroides* spp., and *Ruminococcus* spp. The remaining genera displayed low or undetectable abundance across all groups.

At the genus level, a higher abundance of *Prevotella* spp., was observed in the *L. lactis* and T/*L. lactis* groups compared to the cirrhotic, placebo, and tamsulosin groups. In contrast, the genera *Blautia* spp, *Clostridioides* spp., *Helicobacter* spp., *Bacteroides* spp., and *Ruminococcus* spp., were predominantly detected in the placebo group. Regarding the genera *Colidextrinbacter* spp., *Alloprevotella* spp., *Phascolarctobacterium* spp., *Parabacteroides* spp., *Solobacterium* spp., *Clostridium sensu stricto*, *Clostridia_UCG-014*, *Butyricicoccus* spp., and *Parasutterella* spp., a very low or undetectable abundance was observed in all groups (**Figure 7**).

### 3.3 Effect on hepatic damage

**Figure 8:**
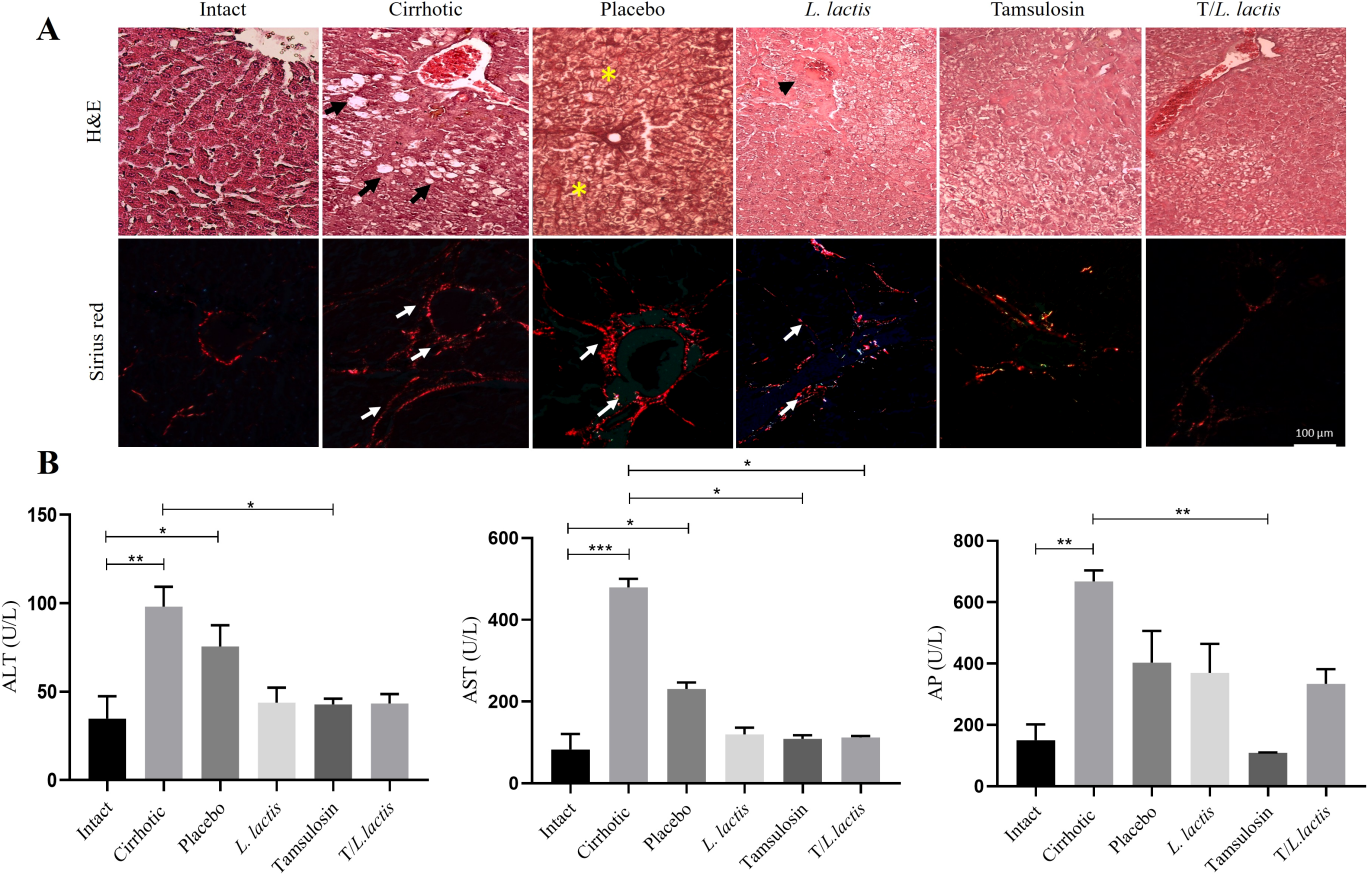
Effects of tamsulosin and *L. lactis* administration on liver morphology and function. **(A)** Analysis of the histological structure of the hepatic parenchyma after treatment, carried out with H&E and Sirius red. White arrow indicated fibrotic area (type I collagen fibers), black arrow indicated macrovesicular and microvesicular steatosis, yellow asterisks indicated regeneration nodules, black arrowhead indicated discontinuous nodules with pyknotic hepatocytes. Magnification, 20x. **(B)** The serum levels of ALT, AST, and AP were used as markers of liver damage. A decrease in ALT and AST levels was observed in the groups that received tamsulosin, *L. lactis*, and their combination. AP levels decreased significantly in the tamsulosin group. The results are presented as mean ± standard error of the mean. Samples from each rat were analyzed in triplicate. The statistical analysis was performed with the Kruskal-Wallis and Tukey post-test methods, where the values of *p < 0.05, **p < 0.01, and ***p < 0.001 were considered significant.

To evaluate the effect of tamsulosin and *L. lactis* administration on liver damage induced by cirrhosis, histopathological analyses and biochemical assessments of hepatic injury markers were performed. Histopathological analysis of liver tissue samples demonstrated distinct morphological differences across experimental groups. In the intact group, hepatic lobules exhibited a well-organized structure characterized by radially arranged hepatocyte cords surrounding the centrilobular vein and a typical distribution of peripheral collagen adjacent to the portal triad. In contrast, the cirrhotic group displayed a markedly disorganized architecture, with hepatocytes showing macrovesicular and microvesicular steatosis. Additionally, prominent type I collagen fibers (visualized via Sirius Red staining) were observed in both perivascular and lamellar regions. The placebo group revealed regenerative nodules and hepatocytes exhibiting ballooning degeneration, with nuclei displaying karyorrhexis and karyolysis; type I collagen distribution resembled the cirrhotic groups. In the groups treated with tamsulosin and *L. lactis*, discontinuous nodules were observed, accompanied by pyknotic hepatocytes and regions of reduced tissue damage relative to the cirrhotic and placebo groups. Notably, the tamsulosin and *L. lactis* (T/*L. lactis*) combined treatment group exhibited significant hepatic parenchyma recovery, with hepatocytes approaching a morphological appearance comparable to the intact group and a diminished presence of collagen fibers. (**Figure 8A**). Regarding the determination of serum markers of liver damage ALT, AST, and AP, cirrhotic animals showed significantly higher ALT(98.0 U/L; *p* < 0.01), AST (479.4 U/L, *p* < 0.001) and AP (667.5 U/L; *p* < 0.01) levels compared to the intact group (ALT: 34.8 U/L, AST: 82.6 U/L, and AP: 150.8 U/L). Rats treated with *L. lactis*, tamsulosin, and the T/*L. lactis* exhibited lower ALT (*L. lactis*; 43.8 U/L, tamsulosin; 42.8 U/L, T/*L. lactis*; 43.3 U/L) and AST (*L. lactis*; 230.8 U/L, tamsulosin; 108.8 U/L, T/*L. lactis*; 112.8 U/L) levels. Currently, AP was significantly reduced only in the tamsulosin group (109.0 U/L, *p* < 0.01) (**Figure 8B**).

## 4 Discussion

In patients with liver cirrhosis, a dysregulation of the liver-gut axis has been found, where splanchnic hyperemia, intestinal dysmotility, and alterations in the intestinal mucosal barrier are observed, as well as changes in gastric function and microcirculation (Tsiaoussis et al., 2015). Jejunal biopsy studies have shown a reduction in the number and length of microvilli, along with thickening, suggesting a decreased absorptive surface area (Wakim-Fleming et al., 2008). In our study, we observed that cirrhotic rats exhibited marked intestinal damage, including epithelial and lamina propria thinning, and significant decreases in villus height and crypt depth. In contrast, the intestinal morphology in the *L. lactis*, tamsulosin, and combination treatment (T/*L. lactis*) groups closely resembled that of the intact controls, with preserved villus height and crypt depth. Consistent with previous findings showing that probiotic administration in TAA-induced cirrhotic rats significantly increased crypt depth, suggesting a protective or restorative effect on intestinal architecture (Jantararussamee et al., 2021). On the other hand, alterations in tight junction (TJ) proteins such as ZO-1, occludin, and claudin have been linked to increased intestinal permeability and systemic endotoxemia (Assimakopoulos et al., 2012). According to our analysis, ZO-1 and occludin expression were observed only in the group treated with *L. lactis*. Some studies have shown that probiotic supplementation can enhance intestinal barrier integrity (Rosenfeldt et al., 2004) by increasing the expression of tight junction proteins such as ZO-1, occludin, and claudins, conferring protective effects against epithelial barrier disruption (Karczewski et al., 2010; Miyauchi et al., 2012; Ukena et al., 2007; Wagnerberger et al., 2013). However, in our study, we did not observe such an effect in the T/*L. lactis* group. Further studies are required to confirm these findings.

Direct evidence regarding the effects of tamsulosin on the intestinal mucosa is limited; existing studies demonstrate that this α1-adrenergic antagonist enhances microcirculatory blood flow in highly vascularized tissues, such as the bladder, within ischemia-reperfusion models (Mizuno et al., 2010). Improved submucosal perfusion has been associated with reduced epithelial injury and accelerated tissue regeneration. Given that cirrhosis-associated intestinal hypoperfusion contributes significantly to epithelial barrier dysfunction (Di Pascoli et al., 2017), it is plausible that tamsulosin exerts protective effects on the intestinal mucosa through similar vascular mechanisms.

A randomized study conducted in male patients with lower urinary tract symptoms (LUTS) and benign prostatic hyperplasia (BPH) demonstrated that after four weeks of treatment with tamsulosin, stool consistency shifted toward softer forms, and constipation scores significantly decreased. These findings suggest that tamsulosin positively influences gastrointestinal transit, likely through the relaxation of intestinal smooth muscle (Matsumoto et al., 2020). Tamsulosin’s α1-adrenergic blockade targets receptors located on vascular and smooth muscle, including intestinal sphincters and smooth muscle layers, which mediate contraction. By blocking these receptors, tamsulosin promotes vasodilation, smooth muscle relaxation, and reduces sympathetic tone. In the setting of cirrhosis, such effects could improve intestinal perfusion and motility, thereby reducing luminal stasis and bacterial overgrowth. Additionally, emerging evidence suggests that tamsulosin may possess anti-inflammatory properties (Alabdali and Ibrahim, 2023), which could further contribute to the preservation of mucosal architecture. Nevertheless, further research is required to confirm these effects in the intestinal context directly.

Bacterial translocation is a key factor in the development of infectious complications in patients with cirrhosis. It contributes to systemic inflammation and worsening of liver function, thereby accelerating disease progression. For this to occur, significant changes must take place, involving modifications in the gut microbiota, damage to the epithelial barrier, and bacterial overgrowth (Simbrunner et al., 2023). The *Enterobacteriaceae* family (*E. coli*, *Klebsiella* spp.), enterococci, and *Streptococcus* spp., are the most observed organisms in bacterial translocation in humans. A study in mice demonstrated that Gram-negative bacteria translocate in large quantities to the MLN, instead of Gram-positive and obligate anaerobic bacteria translocate at much lower levels (Skinner et al., 2020). In our study, we found bacteria in MLNs, spleen, and liver. Specifically, we identified the presence of *Escherichia coli* (*E. coli*), *Klebsiella pneumoniae* (*K*. *pneumoniae*), *Enterobacter agglomerans* (*E. agglomerans*), and *Proteus vulgaris* (*P. vulgaris*) in the cirrhotic and placebo groups, but not in the groups treated with tamsulosin, *L. lactis*, or their combination. Similarly, endotoxin levels and bacterial DNA in blood were found in the cirrhotic and placebo groups. In contrast, endotoxins and bacterial DNA were not detected in the groups treated with *L. lactis*, tamsulosin, and T/*L. lactis* groups. Endotoxemia plays a central role in the pathophysiology of liver cirrhosis, as it reflects the abnormal translocation of bacterial endotoxins such as lipopolysaccharide (LPS) from the gut into the systemic circulation. This process results from increased intestinal permeability and the dysbiosis commonly observed in cirrhotic patients. Probiotic strains such as *Bifidobacterium bifidum* and *Lactobacillus rhamnosus* have been shown to mitigate bacterial translocation and lower systemic LPS levels by enhancing epithelial barrier function (Han et al., 2016). Several studies have shown that *L. lactis* can attenuate LPS-induced endotoxemia by reducing proinflammatory cytokines such as TNF-α and IL-6, preserving intestinal integrity, and limiting microbial translocation (Fu et al., 2024). These results reinforce the potential role of *L. lactis* in modulating host immune responses and preventing endotoxin-mediated complications, particularly in conditions characterized by gut barrier dysfunction, such as liver cirrhosis. Although tamsulosin has not been extensively investigated in intestinal models, the absence of endotoxemia and bacterial translocation in our study suggests a protective effect on the gut barrier. The tamsulosin has not been extensively investigated in intestinal models; the absence of endotoxemia and bacterial translocation in our study suggests an indirect protective effect on the intestinal barrier, as the attenuation of tamsulosin-induced liver inflammation could help reduce systemic inflammatory stress, promoting a more stable environment for the intestinal microbiota (Alabdali and Ibrahim, 2023). Numerous studies have reported alterations in the composition of the intestinal microbiome in various liver diseases, where, in addition to a reduction in species diversity, bacterial overgrowth occurs in the intestine, partly due to decreased intestinal motility (Tsiaoussis et al., 2015). In cirrhotic patients, a decrease in the α-diversity of the gut microbiota has been observed (Oh et al., 2020), and alterations in β-diversity have also been reported, particularly in individuals with NAFLD-related cirrhosis (Behary et al., 2021). These findings suggest that cirrhosis is associated with a less diverse and less stable gut microbiome. In this study, we found that *L. lactis* group exhibited increased α-diversity and a more uniform microbial composition, with a more stable gut environment. The T/*L. lactis* group also showed increased α-diversity, although with less compositional uniformity compared to *L. lactis* group. In addition, the microbial profiles found resembled those of the healthy group. Moreover, both groups exhibited microbial community structures (β-diversity) that more closely resembled those of healthy controls. In contrast, the tamsulosin group displayed reduced α-diversity and greater variability in microbial composition. *Firmicutes* and *Bacteroidetes* are the two most prominent bacterial phyla in the gastrointestinal tract, and their relative abundance, expressed as the *Firmicutes*/*Bacteroidetes* (F/B) ratio, has been recognized as an intestinal homeostasis key indicator. An increased proportion of *Bacteroidetes* and *Proteobacteria* is associated with inflammation (Stojanov et al., 2020). In our study, the untreated and tamsulosin groups exhibited a decrease in *Firmicutes* alongside an increase in *Proteobacteria* and *Bacteroidetes*. In contrast, in the groups receiving *L. lactis* and T/*L. lactis*, we observed a significant increase in *Firmicutes* and *Bacteroidetes*, and a decrease in *Proteobacteria*.

The class *Clostridia* was predominant in the cirrhotic and placebo groups, whereas in the groups that received treatment (with *L. lactis* and T/*L. lactis*), the abundance of *Clostridia* was markedly reduced, accompanied by an increase in the class *Bacteroidia*. Members of this class, particularly the genus *Bacteroides*, are well-known intestinal commensals that protect the gut from pathogenic bacteria and support the nutritional needs of other microorganisms in the gut, playing essential roles in the digestion of complex polysaccharides and the modulation of the immune system (Cheng et al., 2022). At the family level, potentially beneficial autochthonous taxa such as *Lachnospiraceae*, *Ruminococcaceae*, and *Clostridiales XIV* are reduced while potentially pathogenic taxa, including *Staphylococcaceae*, *Enterobacteriaceae*, and *Enterococcaceae* are increased (Bajaj et al., 2015; Chen et al., 2011). We observed a similar decrease in *Lachnospiraceae* and *Ruminococcaceae* in the cirrhotic group. In the group that received *L. lactis*, *Lachnospiraceae* increased, while *Ruminococcaceae* remained unchanged. The significance of *Lachnospiraceae* lies in its production of butyrate, which, along with other short-chain fatty acids, inhibits intestinal inflammation and maintains the intestinal barrier (Vacca et al., 2020). At the genus level, an increase in *Prevotella* spp., was observed in the groups treated with *L. lactis* and T/*L. lactis*, compared to the placebo, tamsulosin, and cirrhotic groups. In contrast, the genera *Blautia* spp., *Clostridioides* spp., *Helicobacter* spp., *Ruminococcus* spp., and *Bacteroides* spp., showed greater relative abundance in the placebo group, which could reflect a microbial imbalance characteristic of cirrhosis without intervention. Other genera such as *Colidextrinbacter* spp., *Alloprevotella* spp., *Phascolarctobacterium* spp., *Parabacteroides* spp., *Solobacterium* spp., *Clostridium sensu stricto*, *Clostridia*_UCG-014, *Butyricicoccus* spp., and *Parasutterella* spp., showed very low or undetectable levels in all groups, with no appreciable differences between treatments. The observed improvements in microbial composition following *L. lactis* administration suggests that this strain may exert beneficial modulatory effects on gut microbiota in cirrhotic conditions. *L. lactis* is known to compete with pathogenic bacteria through the production of antimicrobial peptides such as nisin and the acidification of the intestinal environment, thereby limiting the expansion of *Proteobacteria* and other opportunistic taxa (Bernbom et al., 2006). Additionally, *L. lactis* may indirectly promote the growth of beneficial commensals such as *Lachnospiraceae* and *Prevotella* spp., which are involved in short-chain fatty acid (SCFA) production and immune modulation (Ćesić et al., 2023; Winiarska-Mieczan et al., 2022).

The liver is continuously exposed to gut-derived microbial and metabolic signals through the portal vein, making it particularly sensitive to changes in intestinal homeostasis. The microbial components, such as lipopolysaccharide (LPS), activate hepatic immune responses via pattern recognition receptors like TLR4 (Seki et al., 2007). This leads to increased expression of pro-inflammatory cytokines, including IL-1β and TNF-α, and the activation of hepatic stellate cells (HSCs), contributing to fibrosis (Krenkel and Tacke, 2017). The progression of liver fibrosis is characterized by the distortion of normal hepatocyte structure, the formation of nodules, impaired blood flow, portal hypertension, hepatocellular carcinoma, and liver failure (Aydin and Akcali, 2018). Treatment with tamsulosin, *L. lactis*, and their combination was associated with a substantial recovery of liver architecture, with hepatocyte structures closely resembling those observed in the intact group. Furthermore, biochemical markers of liver injury showed that ALT and AST were lower in the *L. lactis*, tamsulosin, and T/*L. lactis* groups, while AP showed significant reduction only in the tamsulosin group. Tamsulosin has demonstrated antifibrotic and immunomodulatory effects in various experimental models. Its ability to reduce the expression of proinflammatory cytokines such as TNF-α and IL-6 (Ali Hasan and Abdulkhaliq Ibrahim, 2023) as well as profibrotic factors like TGF-β1 (Alrasheed et al., 2023) suggests a beneficial influence on the chronic hepatic inflammatory microenvironment. The use of *L. lactis* in a cirrhosis model prevented steatosis and fibrosis, and reduced serum AST and ALT. Likewise, an immunoregulatory process was observed since a notable decrease in the hepatic expression of IL-1β; this could be due to an increase in intestinal IL-10 as a result of the induced damage, which possibly has a hepatic effect due to the close relationship between the liver and the intestine (Delgado-Venegas et al., 2021).

In conclusion, the results of this study reinforce the crucial role of the gut–liver axis in the pathophysiology of liver cirrhosis and highlight the therapeutic potential of *L. lactis* and tamsulosin. The co-administration of *L. lactis* and tamsulosin promoted the restoration of microbial diversity, improved intestinal barrier integrity, and reduced bacterial translocation and endotoxemia. Complementarily, tamsulosin contributed to the preservation of liver architecture and significantly decreased hepatic enzyme levels. These findings suggest that modulating the gut microbiota and systemic factors through probiotic and pharmacological strategies may represent a promising approach to treat chronic liver diseases. However, additional studies with larger sample sizes are necessary to confirm these effects and clarify underlying mechanisms.

## Data Availability

The original contributions presented in the study are included in the article/supplementary material. Further inquiries can be directed to the corresponding author.
